# Female sex hormones mediate the allergic lung reaction by regulating the release of inflammatory mediators and the expression of lung E-selectin in rats

**DOI:** 10.1186/1465-9921-11-115

**Published:** 2010-08-24

**Authors:** Ana Paula Ligeiro de Oliveira, Jean Pierre Schatzmann Peron, Amilcar Sabino Damazo, Adriana Lino dos Santos Franco, Helori Vanni Domingos, Sonia Maria Oliani, Ricardo Martins Oliveira-Filho, Bernardo Boris Vargaftig, Wothan Tavares-de-Lima

**Affiliations:** 1 Department of Pharmacology, Institute of Biomedical Sciences, University of São Paulo - Av. Prof. Lineu Prestes 1524, São Paulo, 05508-900 - Brazil; 2 Department of Immunology, Institute of Biomedical Sciences, University of São Paulo, Av. Prof. Lineu Prestes 1730, São Paulo, 05508-900 - Brazil; 3 Department of Basic Science in Health, Faculty of Medical Sciences, Federal University of Cuiabá, Av.Corrêa, s/n°, Cuiabá, 78060-900 - Brazil; 4 Department of Biology, Institute of Biosciences, Language Studies and Exact Sciences, São Paulo State University, R. Cristóvão Colombo, 2265, São José do Rio Preto, 15054-000 - Brazil

## Abstract

**Background:**

Fluctuations of estradiol and progesterone levels caused by the menstrual cycle worsen asthma symptoms. Conflicting data are reported in literature regarding pro and anti-inflammatory properties of estradiol and progesterone.

**Methods:**

Female Wistar rats were ovalbumin (OVA) sensitized 1 day after resection of the ovaries (OVx). Control group consisted of sensitized-rats with intact ovaries (Sham-OVx). Allergic challenge was performed by aerosol (OVA 1%, 15 min) two weeks later. Twenty four hours after challenge, BAL, bone marrow and total blood cells were counted. Lung tissues were used as explants, for expontaneous cytokine secretion *in vitro *or for immunostaining of E-selectin.

**Results:**

We observed an exacerbated cell recruitment into the lungs of OVx rats, reduced blood leukocytes counting and increased the number of bone marrow cells. Estradiol-treated OVx allergic rats reduced, and those treated with progesterone increased, respectively, the number of cells in the BAL and bone marrow. Lungs of OVx allergic rats significantly increased the E-selectin expression, an effect prevented by estradiol but not by progesterone treatment. Systemically, estradiol treatment increased the number of peripheral blood leukocytes in OVx allergic rats when compared to non treated-OVx allergic rats. Cultured-BAL cells of OVx allergic rats released elevated amounts of LTB_4 _and nitrites while bone marrow cells increased the release of TNF-α and nitrites. Estradiol treatment of OVx allergic rats was associated with a decreased release of TNF-α, IL-10, LTB_4 _and nitrites by bone marrow cells incubates. In contrast, estradiol caused an increase in IL-10 and NO release by cultured-BAL cells. Progesterone significantly increased TNF- α by cultured BAL cells and bone marrow cells.

**Conclusions:**

Data presented here suggest that upon hormonal oscillations the immune sensitization might trigger an allergic lung inflammation whose phenotype is under control of estradiol. Our data could contribute to the understanding of the protective role of estradiol in some cases of asthma symptoms in fertile ans post-menopausal women clinically observed.

## Introduction

Compelling evidence indicates that female sex hormones play a role not only in healthy airway function but also during inflammation. In the context of airway dysfunction, it is noteworthy that oscillations of sex hormones caused by the menstrual cycle might be linked to asthma deterioration [1,2]. Premenstrual worsening of asthma was described more than 70 years ago [3], with nearly half of asthmatic women exhibiting increased respiratory discomfort during the menstrual period [4]. In addition, there is an exacerbation of asthma symptoms [5] and a decline in lung function [6] at the luteal phase of the cycle, when estradiol levels decrease. Conversely, the frequency and severity of asthma deterioration reduces when serum levels of estradiol are high, as observed after exogenous estradiol, oral contraceptives usage and during ovulation [7]. In fact, forced expiratory volume and vital capacity are higher during early luteal phase, when estradiol and progesterone levels are high [8]. Overall, these data reinforce the inverse correlation between female sex hormone levels and deterioration of asthma symptoms. Of interest is the data reporting that menstrual cycle, contraceptive usage and hormonal replacement therapy account for asthma deterioration in women [9-14], a fact whose mechanisms are yet unclear.

Asthma is a Th_2_-lymphocytes mediated disease and interestingly the fluctuations of circulating female sex hormones during the menstrual cycle lead to a significant increase of cytokines associated to a Th2-type of response [15]. Accordingly, IL-4 production by CD4^+ ^T cells is affected by cyclical variations of circulating estradiol levels [16,17]. Yet, lymphocytes of asthmatic women did not express normal β_2 _adrenoceptors, a fact that might be related to increased bronchial responsiveness [18].

We have recently demonstrated that OVA sensitization 7 days after ovariectomy widely reduces IL-5 and eosinophil recruitment to the lungs in the murine asthma model [19]. Besides, using the same model, we have also observed that antigen-induced mast cell degranulation is somehow impaired [20]. It is worthy to mention that these cells are very involved in acute asthma attacks [21,22]. In this 5 context, it has been demonstrated that estradiol also facilitates histamine release after antigen challenge [23], where estradiol α-receptor seems to play a role using a non-genomic pathway [24]. Besides, estradiol upregulates cellular recruitment and cytokine release into lungs after antigen challenge in rats [20,25].

Using a rat model of allergic lung inflammation, we have also demonstrated that antigen sensitization 7 days after ovaries removal culminates in a drastically decreased cell recruitment into lungs after antigen challenge [25]. Similarly, allergic response triggered in intact females upon tamoxifen treatment was found also reduced [26]. Tamoxifen has a triphenylethylene structure (C_26_H_29_NO) wich directly blocks the effect of estrogen on tissue, preventing estrogens from binding and activating the cell [27]. Thus, as tamoxifen is a well-recognized estradiol receptor modulator these data reinforce the involvement of sex hormones, notably estradiol, over the immune allergic response. Overall, we [20,25,19] and others [28] have observed that estradiol displays pro-inflammatory actions, such as the already mentioned mast cell degranulation and bronchial hyperresponsiveness. On the other hand, estradiol is also reported to improve lung function during perimenstrual asthma [29], ameliorating lung inflammation and decreasing lung remodeling in murine asthma model [30]. Taking these evidences into account, it is noticeable the conflicting picture regarding the effects of sex hormones on asthma [31,27]. Thus, the situation deserves a better understanding on the role of sex hormones in inflammatory mechanisms underlying lung inflammation.

In the present study, we hypothesize that the profile of circulating sex hormones during antigen sensitization exerts a pivotal role on the scores of allergic lung inflammation. To examine that we investigated the magnitude of allergic lung inflammation and the release of inflammatory mediators in female rats sensitized to antigen 1 day after ovariectomy.

## Materials and methods

### Animals

Female Wistar rats (180-220 g) from the Institute of Biomedical Sciences animal facilities were used throughout. Animals were housed in groups of five rats per cage in a light- and temperature-controlled room (12/12-h light-dark cycle, 21 ± 2°C) with free access to food and water. All experiments were approved by the local Animal Care Committee.

### Ovariectomy (OVx)

Rats were anesthetized with an intraperitoneal injection of ketamine-xylazine (100 and 20 mg/kg, respectively). Upon laparotomy the ovaries were removed free from adherent tissue. The surgical wound was sutured and animals received a single dose of Pentantibiotic^® ^(570 mg/kg) by intramuscular route. Vaginal smears, quantification of the uterine weight and determination of the circulating levels of estradiol and progesterone, were used in order to assess the effectiveness of OVx. Similarly operated rats except for ovaries removal were used as the sham-operated controls (Sham-OVx group). A non-manipulated group of female rats (Basal group) was used to obtain the normal basal values of all parameters studied.

### Sensitization and antigen challenge

**Figure 1:**
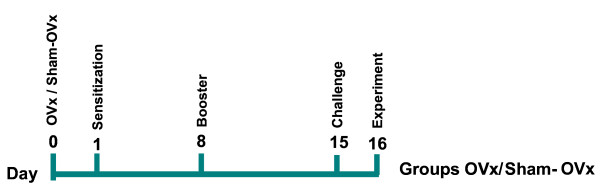
**Experimental design of ovariectomy (OVx) and ovalbumin-(OVA) sensitization**. Anesthetized rats were subjected to ovariectomy (OVx) and at the indicated day 1 were ovalbumin (OVA) sensitized. Control group consisted of rats submitted to similar manipulations excepting the ovaries removal (Sham-OVx). Sensitized rats were boosted with OVA (Day 8), and the OVA-challenge performed at day 15. The rats were euthanized at day 16 (see material and methods for more details).

One day after ovaries removal, (OVx) or Sham-OVx rats were sensitized by an intraperitoneal injection of a suspension of 10 μg of OVA with 10 mg aluminum hydroxide. One week later, rats were boosted subcutaneously with 10 μg of OVA dissolved in phosphate buffer solution (PBS). Two weeks after first sensitization, rats were subjected to a single 15-min exposure of aerosolized OVA (1% in PBS) using an ultrasonic nebulizer device (Icel, São Paulo, Brazil) coupled to a plastic inhalation 7 chamber (18.5 × 18.5 × 13.5 cm) and labeled as "allergic". Rats were euthanized 24 h after challenge by sectioning of the abdominal aorta under deep chloral hydrate anesthesia (> 400 mg/kg ip) (Fig. 1).

### Bronchoalveolar lavage (BAL) and cell countings

A total volume of 40 ml (twice, 20 ml) of PBS was injected into the rat lungs by tracheal route [25]. BAL fluid was collected and centrifuged (170 *g*, 10 min) and the cell pellet resuspended in 1 ml PBS. Aliquots of cell suspension (90 μl) were stained with 10 μl of 0.2% crystal violet and total cells quantified by microscopy using Neubauer chamber. Differential cell countings (neutrophils, eosinophils, and mononuclear cells) were carried out using standard morphological methods after cytospin processing and Rosenfeld's dye staining.

### Blood leukocytes and bone marrow cell counts

Peripheral blood leukocytes and bone marrow cells were quantified in samples from the tail vein and from aliquots of medullary femural lavage (FL) respectively [20]. Blood aliquots were diluted (1:20) in Türk fluid (3% acetic acid) and cells of FL were resuspended in PBS (1 ml), stained with crystal violet (0.2%). The quantitative analyses of cells were performed in blood smears and FL samples stained with Rosenfeld's dye [20]

### Determination of TNF-α, IL-10, LTB_4 _and nitrites levels

Total cells recovered from BAL were suspended in 10% fetal bovine serum (FBS)-enriched, RPMI-1640 culture medium (1000 μL). The trypan blue exclusion test was employed to determine cell viability. Aliquots (500 μl) containing 2 × 10^6 ^cells/ml were platted into 24-well plastic microplates under a 5% CO_2_-95% O_2 _atmosphere at 37°C. Aliquots of supernatants were collected 24 h later and stored at -80°C. TNF-α activity was evaluated by a cytotoxicity assay using L-929 cells as described previously [32]. TNF-α titer (U/ml) is defined as the reciprocal of the dilution that induces 50% of lysis of L-929 cells. LTB_4 _and IL-10 concentration were quantified in samples of BAL and FL cells using ELISA kits purchased from R&D Systems (Minneapolis, MN). Detection limit was 7.8-500 pg/ml for LTB_4 _and 62.5-4000 pg/ml for IL-10. Nitrites concentration was determined by colorimetric assay (540 nm) in supernatant of cellular cultures of total BAL and the FL using the Griess reaction [33]. In brief, 2.0 × 10^6 ^cells/ml BAL or the FL were distributed in plastic microplates of 24 wells containing RPMI-1640 culture medium (1 ml) supplemented with 10% FBS. The reaction was performed adding 50 μl of BAL or FL cells culture supernatant in plastic microplates (96 wells) containing the equal volume of Griess reagent at room temperature for 10 min. Aliquots of supernatants of BAL or FL cultured cells from non-manipulated rats (Basal group) were used as controls. The optical density was obtained using automatic ELISA reader (Bio-Tek Instruments^®^), and the concentration of nitrites were determined using previously established standard curve of NaNO_2 _(5-60 μM).

### Estradiol and progesterone quantification

Blood samples were collected from the orbital plexus of anesthetized rats before immunization (day 0, corresponding to 1-day OVx), at the booster (day 7, corresponding to 8-day OVx), at the challenge (day 14, corresponding to 15-day OVx), and 24 h after antigen challenge (day 15, corresponding to 16-day OVx). Aliquots of blood were centrifuged (170 g, 10 min), and the resulting sera were stored at -70°C until further analyze. The hormones were determined using ELISA kits (Diagnostic Products, Los Angeles, CA). Detection limits were 0.011-0.025 pg/ml for estradiol and 0.009-0.020 ng/ml for progesterone.

### Immunohystochemistry for lung E-Selectin expression

Animals were euthanized as described, lungs exposed and filled by the trachea with 10 ml of tissue freezing medium (OCT - Leica Instruments, Wetzlar, Germany) dilute 1:3 in distilled water. Trachea and lungs were then removed and small fragments immersed in hexane in liquid nitrogen. Samples were submitted to 8 μm sections and fixed in acetone for 10 min. All sections were washed 3 times in PBS during 30 min and then kept in hydrogen peroxide (3%) for 10 min at room temperature for endogenous peroxidase activity blocking. Slides were washed again 3 times in PBS and then blocked for unspecific binding with BSA (10% - 30 min) and Super block solution (Thermo Scientific Pierce Protein Research Products, Rockford, USA) for 2 h at room temperature in chambers with controlled humidity. Afterwards, all slides were incubated with anti-E-selectin at 1:50 in PBS/Tween 20 (0.3%, 4°C overnight). Following incubation, samples were washed in PBS and incubated with biotinilated secundary antibody mouse anti-Rat IgG (1:1000) for 1 h at room temperature. After that, slides were washed in PBS and incubated with streptavidin- peroxidase (Vectastain ABC Kit - Vector Laboratories, CA, USA) for 1 h at room temperature. Assay was then performed with 0.5 mg/ml of diaminobenzidine (DAB - BioGenex, CA, USA) and hydrogen peroxyde 0.06% for visualization. Samples were counter-stained with hematoxilin eosin and submitted to dehydration with ethanol (70% - 90% -95% - 100%), diafanization (Xylol - Merck, Sao Paulo, Brazil) and then covered with Entellan (Merck, Sao Paulo, Brazil) and coverslip. All analyses were performed comparing at least 3 samples from the same animal for a total of 3 animals per group. Bronchial regions were selected and the antibody stained regions were determined by imaging software analysis KS-300 (Carl Zeiss, Jena, Germany). Results are expressed as staining density at arbitrary units.

### Pharmacological treatments

Twenty-four hours prior to OVA challenge, groups of OVx allergic rats were treated with a single subcutaneous injection of 17β-estradiol (280 μg) or progesterone (200 μg). Controls consisted of OVx allergic rats subjected to injection with the corresponding volumes of the hormone vehicles (corn oil for 17β-estradiol or distilled water for progesterone).

### Statistical analysis

Data are presented as mean ± S.E.M. Comparisons between groups were made by one-way ANOVA followed by Newman-Keuls post test. The 4.0 version of GraphPad InStat Software was used for these purposes. Values of *P *< 0.05 were considered significant.

## Results

### Circulating levels of estradiol and progesterone at the phases of OVA-immune sensitization

Table 1 shows that at the day of OVA-sensitization, serum levels of estradiol and progesterone of 1-day OVx rats were similar to those found in estrous. At the booster day (8-day OVx), the serum levels of estradiol and progesterone drastically reduced comparatively to those found at the OVA-sensitizing day. At the challenge (15-day OVx) and at the experiment day (16-day OVx), the concentration of estradiol and progesterone did not differ from those found at the booster day (8-day OVx). In a parallel set of experiments, we observed that vaginal smears of OVx rats were morphologically compatible with diestrous phase. In addition, the uterus weight of OVx rats significantly reduced compared with that of Sham-OVx rats (data not shown).

**Table 1:** Serum levels of estradiol and progesterone before and after OVx and the effects of treatments with estradiol or progesterone before albumin challenge.

		Estradiol (pg/ml)	Progesterone (ng/ml)
**Sham OVx**	*Estrous*	15.6 ± 3.0	10.6 ± 1.2
	*Diestrus*	8.3 ± 1.5	21.3 ± 1.2
	*Metaestrus*	15.2 ± 1.0	21.0 ± 0.2
	*Proestrus*	34.3 ± 4.6	4.0 ± 0.2
			
**OVx**	*At sensitization (1-day OVx)*	11.4 ± 1.5	13.8 ± 3.0
	*At booster (8-day OVx)*	5.9 ± 0.3	4.8 ± 1.0
	*At challenge (15-day OVx)*	5.7 ± 1.8	3.9 ± 0.3
	*At experiment (16-day OVx)*	5.2 ± 1.5	3.2 ± 0.3

Values are means ± SE of radioimmunoassay data from 5 rats/group. Orbital plexus blood as taken under deep anesthesia from sham OVx allergic and Sham-Ovx allergic rats at various time points corresponding to the experimental steps.

### Repercussion of ovaries removal (OVx) to OVA-induced allergic lung inflammation

**Figure 2:**
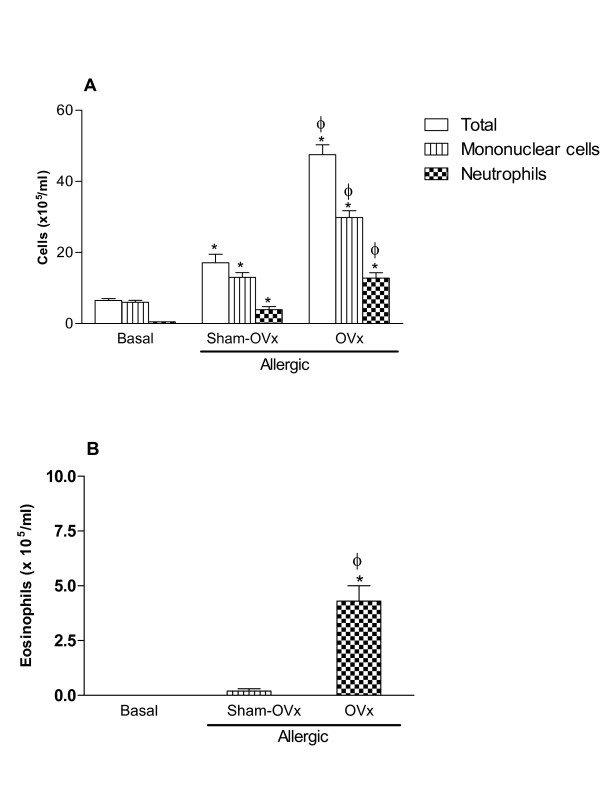
**Total mononuclear cells and neutrophils (A) and eosinophil counts (B) in bronchoalveolar lavage (BAL) fluid of allergic rats (Sham-OVx and OVx)**. Basal values were obtained from nonmanipulated rats. Data are means ± SE from 5-8 experiments. *P < 0.05 compared with the basal group; ϕ P < 0.05 compared with the Sham-OVx allergic group.

As demonstrated in Fig 2, ovaries removal 1 day prior to OVA-sensitization was associated with an increased number of cells collected in BAL (OVx allergic) when compared with the cells collected in BAL of Sham-OVx allergic rats. Moreover, the number of mononuclear cells, neutrophils (Fig 2A) as well as eosinophils (Fig 2B), was significantly increased in both groups of allergic rats (Sham-OVx and OVx) but such increase was more pronounced in OVx allergic rats.

### Changes in blood leukocytes and bone marrow cells counting in OVx-allergic rats

**Figure 3:**
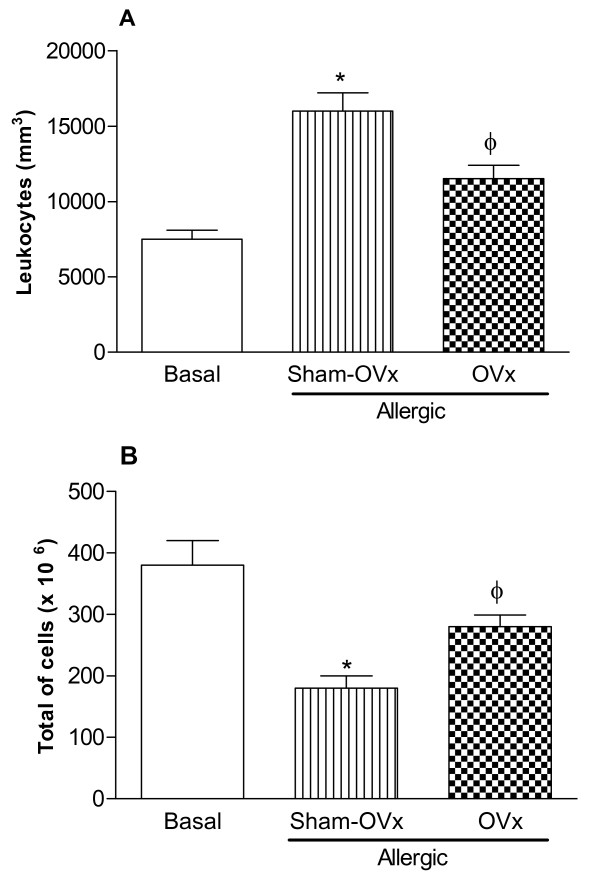
**Number of cells in peripheral blood (A) and in bone marrow (B) from allergic Sham-OVx and OVx rats**. Basal values were obtained from nonmanipulated rats. Data are means ± SE from 5-8 experiments. *P < 0.05 compared with the basal group; ϕ P < 0.05 compared with the Sham-OVx allergic group.

Once we observed a significant increase in cell countings in BALs from OVx allergic rats, we sought to evaluate whether OVx might modify the circulating cell profile in blood and bone marrow. Fig. 3 shows that allergic Sham-OVx and OVx allergic rats had increased countings of circulating leukocytes (Fig. 3A) whereas the reduced number of bone marrow cells were not affected by ovaries removal (Fig. 3B). Interestingly, OVx allergic rats reduced the blood leukocytes counting and increased that of bone marrow cells comparatively to those found in the Sham-OVx allergic group.

### Effects of estradiol and progesterone treatments

#### BAL cells count

**Figure 4:**
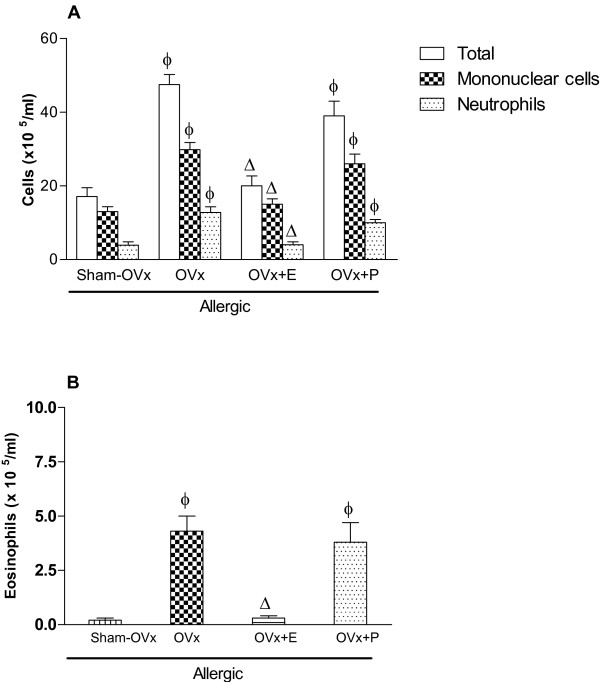
**Involvment of estradiol and progesterone in total cells, mononuclear cells and neutrophils (A) and eosinophils (B) recovered in BAL of rats subjected to allergic lung inflammation**. Estradiol (280 μg s.c., single dose) and progesterone (200 μg s.c., single dose) replacement was performed in OVx rats 24 h before the antigen challenge. Data are means ± SE from 5-8 experiments. ϕ P < 0.05 compared with the Sham-OVx allergic group; Δ P < 0.05 compared to the OVx group.

As shown in Fig 4A, estradiol treatment of rats previously to OVA-challenge prevented the increased cell counting of BAL collected from OVx allergic rats as compared to non-treated OVx allergic rats. In addition, an intense reduction of eosinophil counting in BAL of OVx allergic rats was also found after estradiol treatment (Fig. 4B). On the other hand, progesterone treatment of rats did not modify the augmented number of cell counting of BAL of OVx allergic rats caused by OVA-challenge.

#### Blood leukocytes and bone marrow cells count

**Figure 5:**
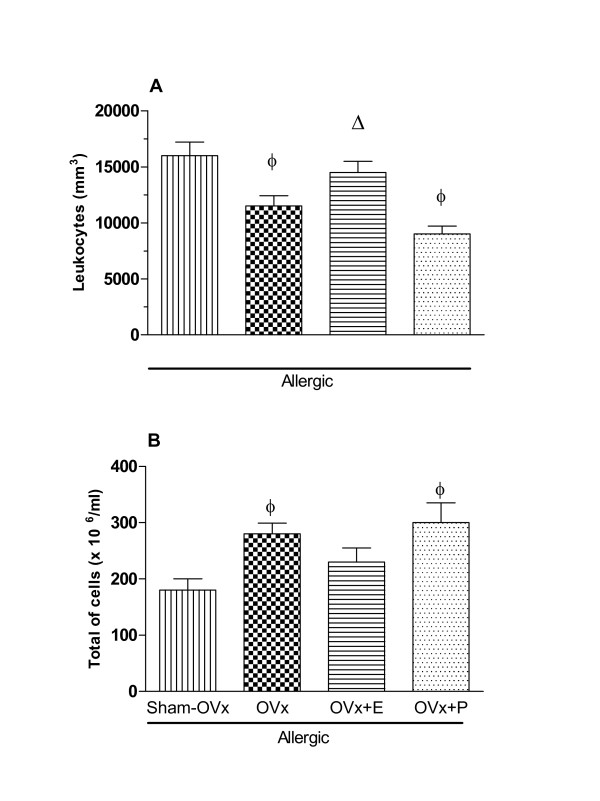
**Involvment of estradiol and progesterone in total cells in peripheral blood (A) and in bone marrow (B) from Sham-OVx and OVx allergic rats**. Estradiol (280 μg s.c., single dose) and progesterone (200 μg s.c., single dose) replacement was performed in OVx rats 24 h before the antigen challenge. Data are means ± SE from 5-8 experiments. ϕ P < 0.05 compared with the Sham-OVx allergic group; Δ P < 0.05 compared to the OVx group.

Fig 5A shows that estradiol, but not progesterone, treatment of OVx allergic rats prevented the decreased blood cell counting as found in blood of non-treated OVx allergic rats. In contrast, estradiol and progesterone treatments did not alter the bone marrow cells counting (Fig 5B).

### Tumor necrosis factor-α (TNF-α) and interleukin 10 (IL-10) release by cultured cells

**Figure 6:**
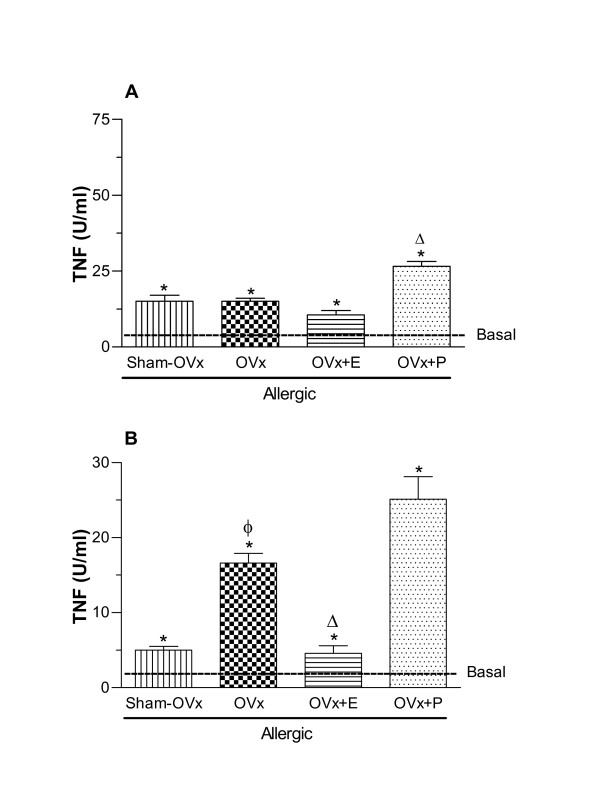
**TNF-alpha released by BAL (A) and bone marrow cells (B) 24 h after in vivo antigen challenge of allergic rats (Sham-OVx and OVx)**. Rats of OVx allergic groups were treated with estradiol or progesterone before the antigen challenge. Basal values were obtained from nonmanipulated rats. Data are means ± SE from 5-8 experiments. *P < 0.05 compared with the basal group; ϕ P < 0.05 compared with the Sham-OVx group; Δ P < 0.05 compared with the untreated OVx allergic group.

TNF-α is a proinflammatory cytokine which has been implicated in many aspects of the airway pathology in asthma. It directly induces histamine release from mast cells and also potentiates its cytokine secretion. Moreover, TNF-α is particularly important in the development of airway hyperresponsiveness [34]. Our data revealed that TNF-α levels released by BAL-cultured cells of Sham-OVx and OVx allergic rats were significantly higher than those found in BAL cells of the Basal group. After progesterone treatment of OVx allergic rats, TNF-α levels generated by BAL cultured cells were significantly increased as compared to their untreated counterparts, whereas estradiol treatment did not change the TNF-α secretion by BAL cells (Fig. 6A). Bone marrow cells (Fig. 6B) from estradiol-treated OVx allergic rats significantly decreased the concentration of TNF-α regarding the cells from untreated OVx allergic rats, while progesterone treatment did not change the TNF-α generation.

**Figure 7:**
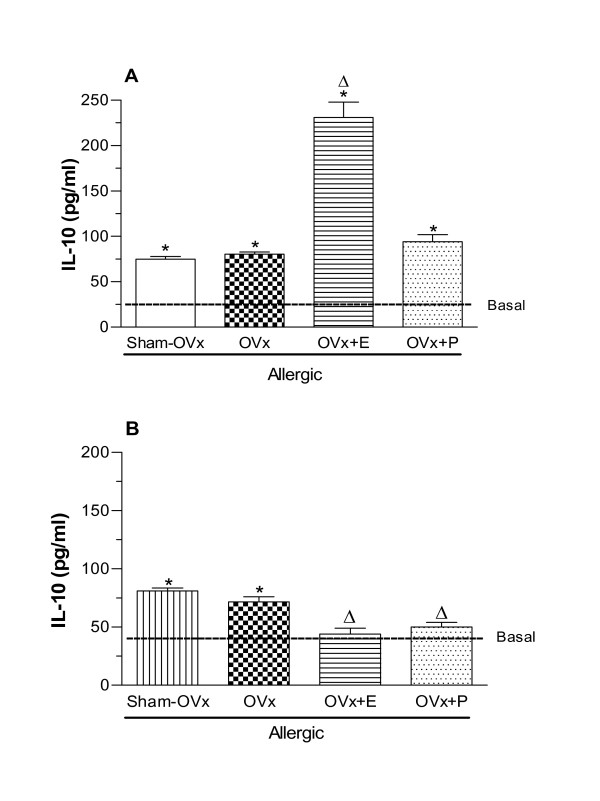
**IL-10 released by BAL (A) and bone marrow cells (B) 24 h after in vivo antigen challenge of allergic rats (Sham-OVx and OVx)**. Rats of OVx allergic groups were treated with estradiol or progesterone before the antigen challenge. Basal values were obtained from nonmanipulated rats. Data are means ± SE from 5-8 experiments. *P < 0.05 compared with the basal group; ϕ P < 0.05 compared with the Sham-OVx group; Δ P < 0.05 compared with the untreated OVx allergic group.

IL-10 is anti-inflammatory cytokine exerting it effects as potent inhibitor of monocyte/macrophage function and the production of pro-inflammatory cytokines [35]. In this context, we sought to evaluate the IL-10 produced by BAL-cultured cells from Sham-OVx and OVx allergic rats. Our data showed that the level of this cytokine did not differ between the Sham -OVx and OVx rats. On the other hand, estradiol treatment of OVx-rats markedly increased the generation of IL-10 whereas progesterone was ineffective to alter such levels (Fig. 7A). Regarding bone marrow cells, estradiol and progesterone treatments of rats significantly decreased the levels of IL-10 of OVx allergic as compared to untreated OVx allergic rats. By contrast, irrespective of ovaries removal, IL-10 levels generated by bone marrow cells were similar among the groups (Fig 7B).

### Leukotriene B_4 _(LTB_4_) and nitrites production by cultured cells

**Figure 8:**
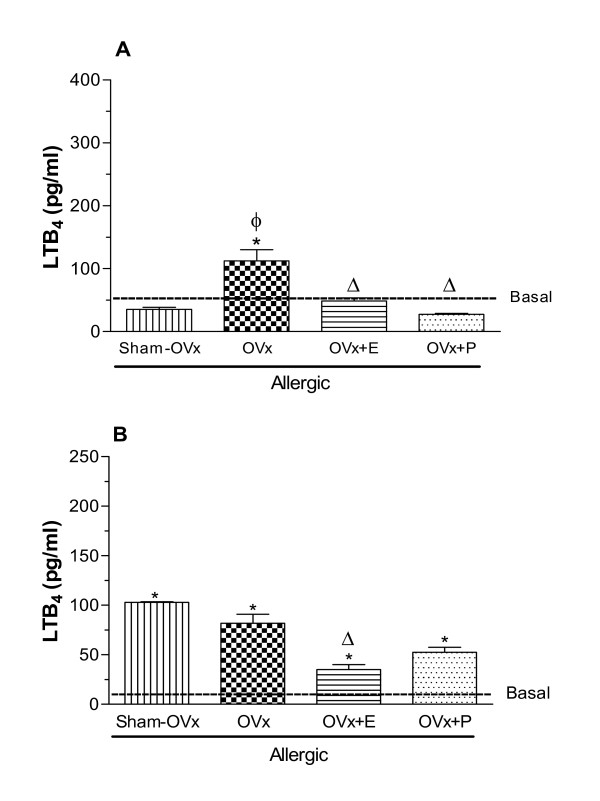
**LTB_4 _released by BAL (A) and bone marrow cells (B) 24 h after in vivo antigen challenge of allergic rats (Sham-OVx and OVx)**. Rats of OVx allergic groups were treated with estradiol or progesterone before the antigen challenge. Basal values were obtained from nonmanipulated rats. Data are means ± SE from 5-8 experiments. *P < 0.05 compared with the basal group; ϕ P < 0.05 compared with the Sham-OVx group; Δ P < 0.05 compared with the untreated OVx allergic group.

Leukotrienes are widely known for their properties as potent bronchoconstrictors, ability to increase airway responsiveness, vascular permeability and mucus production [36]. Its activities include chemotaxis of neutrophils and eosinophils, aggregation of neutrophils, and enhanced expression of complement receptors on granulocytes. Similarly, nitric oxide is also thought to be involved in asthma, including tissue repair, vasodilation and inflammation as extensively reviewed [36,37]. Due to these features, we also decided to quantify LTB_4 _and nitrites levels in samples of BAL-cultured cells. Cells of OVx allergic rats released higher amounts of LTB_4_, an effect which was significantly prevented by estradiol or progesterone treatments before OVA- challenge (Fig. 8A). In contrast, bone marrow cells from Sham OVx allergic rats significantly increased LTB_4 _levels compared to bone marrow cells of basal group. Although OVx did not modify LTB_4 _release by bone marrow cells, estradiol treatment of rats decreased the LTB_4 _quantification whereas progesterone was ineffective (Fig. 8B).

**Figure 9:**
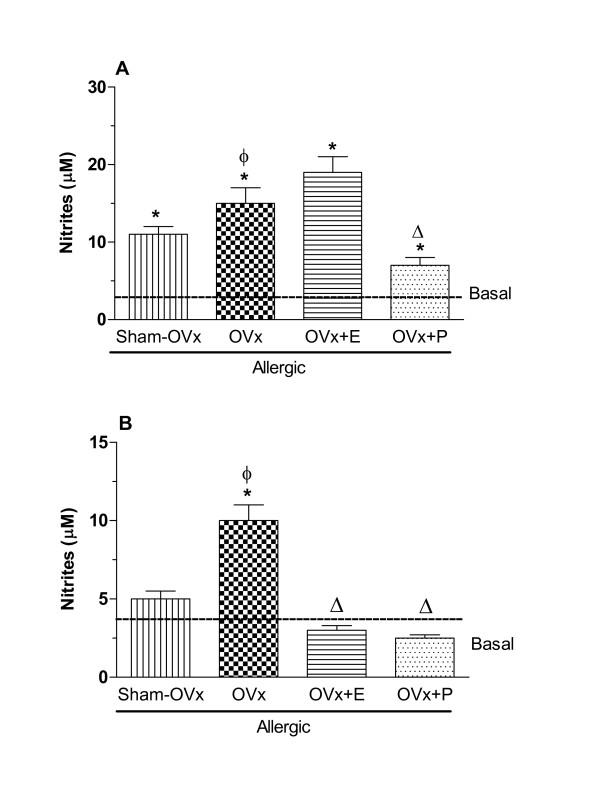
**Nitrites released by BAL (A) and bone marrow cells (B) 24 h after in vivo antigen challenge of allergic rats (Sham-OVx and OVx)**. Rats of OVx allergic groups were treated with estradiol or progesterone before the antigen challenge. Basal values were obtained from nonmanipulated rats. Data are means ± SE from 5-8 experiments. *P < 0.05 compared with the basal group; ϕ P < 0.05 compared with the Sham-OVx group; Δ P < 0.05 compared with the untreated OVx allergic group.

OVx increased the concentration of nitrites released by cultured-BAL cells after OVA-challenge (OVx-allergic) but such levels were not affected by estradiol treatment. On the other hand, progesterone treatment effectively decreased the nitrites release caused by OVA-challenge (Fig. 9A). Nitrites levels released by bone marrow cells were significantly increased by OVx in allergic rats and were reduced by estradiol and progesterone treatments (Fig. 9B).

### Regulatory role of sex hormones on the lung expression of E-selectin in allergic rats

**Figure 10:**
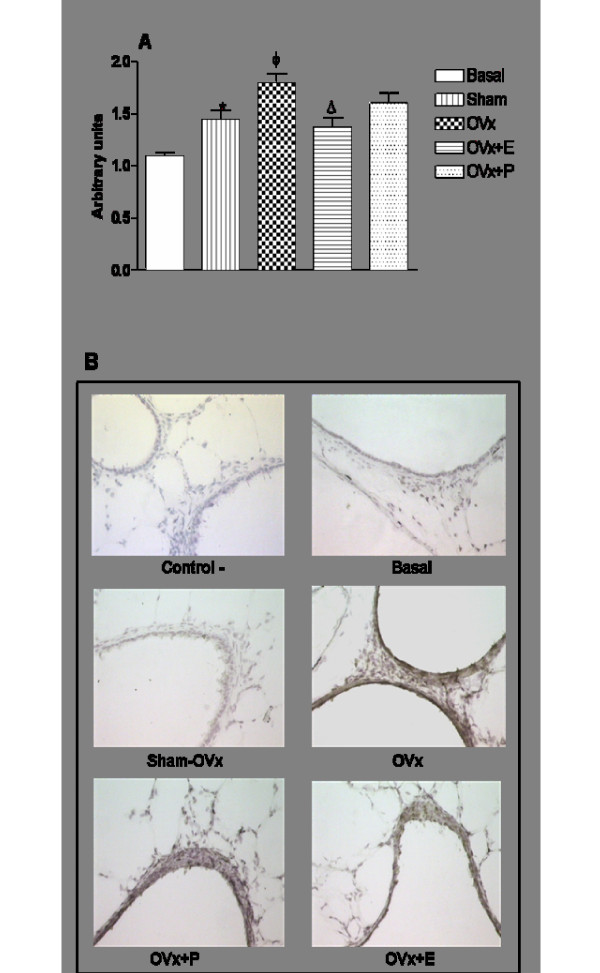
**Immunohystochemistry for lung E-Selectin expression of cells from BAL of allergic rats (Sham-OVx and OVx)**. Rats of OVx allergic groups were treated with estradiol or progesterone before the antigen challenge. Basal values were obtained from nonmanipulated rats. Data are means ± SE from 5-8 experiments. *P < 0.05 compared with the basal group; ϕ P < 0.05 compared with the Sham-OVx group; Δ P < 0.05 compared with the untreated OVx allergic group.

Ovaries removal significantly increased E- selectin expression in lungs of allergic rats when compared to lungs of Sham OVx- allergic rats. Lungs of OVx-allergic rats upon estradiol treatment decreased the E-selectin expression to levels close to those found in lung of Sham-OVx allergic rats. In contrast, progesterone treatment did not exert effects on the E-selectin expression of lung of OVX-allergic rats (Fig. 10A). Representative pictures of immunohistochemical lung expression of E-selectine in OVx allergic rats and their matched controls are depicted in Fig. 10B.

## Discussion

The scope of this study holds on to the fact that women at the perimenstrual period and those at postmenopause upon hormonal replacement therapy show worsened asthma symptoms. On the other hand, asthmatic fertile women under oral contraceptive usage display reduced Th2 responses, reduction in asthma symptoms, improvement of lung function, and/or reduction in medication use in women taking hormonal preparations [9,11,12,14]. Overall, it is clear that the outcome of the allergic response differs whether under oscilating or decreased levels of female sex hormones. These evidences highlight a causal link between the oscillations of sex hormones and the overall allergic response. However, the role of the fluctuations of sex hormones on the inflammatory aspects of allergic lung disease is yet unclear.

Here, using a well-established rodent model of allergic lung disease we have reported the contrast between the lung inflammatory response of ovariectomized (OVx) allergic rats compared to found in Sham-OVx allergic animals. We observed that rats upon ovaries removal 1 day prior to OVA-sensitization when subjected to OVA-challenge developed a more robust increase of eosinophils, neutrophils and monocytes cells into lung. In addition, an increased E-selectin expression in lungs was also found. In the context of cell trafficking, caused by allergic challenge, the ovaries removal increased the number of bone marrow cells and decreased that of peripheral blood leukocytes. Altogether, these events might justify the accumulation of inflammatory cells into lung after OVA challenge in OVx rats.

As the ovaries removal of rats was carried out previously to OVA-immune sensitization then these data allowed us to recognize, as reported earlier [38], a putative role of female sex hormones on the mechanisms involving the adaptative immunity response and immune system cells, notably on those of allergic lung inflammation. An important point of our model of allergic lung inflammation is that the OVA-immunization was performed 1 day after the ovaries removal, whereas the allergic immune response (OVA challenge) was triggered two weeks later. Therefore, the decreasing of circulating levels of estradiol and progesterone after ovaries removal occurred during the OVA-immune sensitization process. As the percentage of bronchial mast cells degranulation of OVx allergic rats after OVA challenge was similar to observed in sham OVx allergic rats and keeping in mind that IgE exerts a crucial role on the immune mechanisms involvingimmune mast cell degranulation, we concluded that synthesis of anafilatic antibodies was not affected by ovaries removal. We observed that cells collected of BAL and of bone marrow of OVx allergic rats significantly increased the release of inflammatory mediators such as LTB_4_, NO and TNF-α. Thus, female sex hormones not only mediated the cell influx but also modulated the functional activity of phagocytes recruited by the allergic challenge.

As the two major ovarian hormones are estradiol and progesterone and ovariectomy augmented the number of inflammatory cells into lung after the allergic challenge, then we treated OVx rats with estradiol or progesterone previously to OVA challenge. Our data showed that estradiol but not progesterone prevented the excessive cell infiltration into the lung and also reestablished the number of blood circulating leukocytes at the levels of sham OVx allergic rats. Estradiol exerts its effects acting on inflammatory cells bearing α and β receptors [39]. In the present study estradiol was administered to OVx rats 24 h before the OVA-challenge, a fact that preclude any speculation regarding its genomic and non genomics effects on lung inflammation control. On the other hand, estradiol could be considered as endogenous regulator of mechanisms associated to cell adhesion and consequently might interfere with functional activity of leukocytes. Indeed, E-selectin expression of lung was decreased in estradiol treated OVx allergic rats. Considering the involvement of E-selectin on the leukocyte accumulation at the inflammatory site [40], then the magnitude of allergic lung inflammatory is endogenously controlled by estradiol. Interestingly, estradiol prevents artery wall thickness by mechanism involving a diminished E-selectin expression in postmenopausal women [41]. Thus, our data reveal that during the allergic lung inflammation, estradiol downregulated the cell recruitment likely preventing the cell adhesion. As estradiol exerts a protective role of endothelium we hypothesized that it could be a therapeutic tool for allergic lung diseases as developed in this study. Indeed, experimental evidences suggest that estradiol prevent the lung inflammatory aspects of asthma. Moreover, in a non allergic model estradiol protects the lung from inflammation caused by innate immune response such as induced by hemorrhagic shock and endotoxin [42].

Next, we investigated the interaction of sex hormones on functional activity of phagocytes measuring the levels of inflammatory mediators in supernatants of BAL and bone marrow cultured cells from OVx allergic rats. Our data indicated that TNF-α generation in lung cells was up regulated by progesterone while estradiol prevented the release of this cytokine by the bone marrow cells. Interestingly, there are studies indicating that ovariectomy reduces serum levels of TNF-a [43]. Nevertheless, our study was carried out using ovariectomized allergic rather than naïve animals. In this context TNF-a increase shown by our data is thus considered a phenomenon that is secondary to antigen sensitization and challenge. In addition, IL-10 levels increased in lung cells of OVx allergic rats upon estradiol treatment. Speculatively we infer that a high level of IL-10 was associated to TNF generation in order to opposite the inflammatory effects of TNF on lung inflammation. In parallel, LTB_4 _release by lung cells was prevented by estradiol and progesterone. Along with TNF-α and LTB_4 _which share pro-inflammatory activity [44] and NO exerts pro and antinflammatory effects [45], our data revealed that sex hormones interfered with NO generation by cultured cells. We found that the high levels of nitrites in cultured cells of OVx allergic rats were a progesterone-mediated event. However, it is yet unclear how NO generated in the lung environment could decrease the leukocyte endothelium interaction. In this context, as progesterone treatment decreased NO generation, its effects could account for the worsening of the allergic lung inflammation. In fact, decreasing NO, progesterone effects could increase cell adhesion and recruitment. On the other hand, estradiol increasing IL-10 levels and decreasing those of TNF-α and LTB4 could prevent cell mobilization into lung after OVA-challenge. Noteworthy, estradiol protects airways of experimental hyperresponsiveness since it increases airways AChE activity as well as prevents mucus and collagen deposition in asthma models [46,30].

Our data also showed that progesterone did not modify the cell influx into the lung of OVx allergic rats, but was effective to increase the levels of TNF-α, and decrease those of LTB_4 _and NO released by BAL-cultured cells. Analyzing the profile of inflammatory mediators released by recruited cells, estradiol effects might be considered as a lung inflammatory deterrent while progesterone could be interpreted as a lung inflammation accelerator. Interestingly, despite the mobilization of bone marrow cells towards to inflammatory site represents a relevant step of defensive response against a noxious stimulus [47] our data revealed that sex hormones are not the regulators of bone marrow cell traffick.

It is worthy to mention that the marked allergic lung inflammation observed here was found in OVA sensitized rats 1 day after ovaries resection (OVx-1 protocol). On the other hand, previously, we demonstrated that rats OVA-sensitized 7 days after OVx (OVx-7 protocol) when exposed to OVA challenge markedly blunted the cell influx into lung [25], which was reverted by estradiol treatment. Thus, in contrast to data utilizing OVx-7 protocol [19,20], in the present study (OVx-1 protocol) estradiol clearly showed a protective role in allergic lung inflammation. The profile of circulating sex hormones at the time of OVA sensitization and those at the time of OVA challenge could justify the polarized effects of sex hormones on the allergic lung inflammation among OVx-1 and OVx-7 protocols. Indeed in OVx-1 protocol circulating levels of estradiol and progesterone, at the time of OVA-sensitization and challenge, were compatible with estrous and diestrous phases respectively (Table 1). On the other hand, in the OVx-7 protocol the OVA-sensitization and challenge were performed at diestrous phase [20]. Thus, we suggest that estradiol and progesterone levels at the period of the first contact of the organism with the antigen could determine the phenotype of allergic lung inflammation. As estrogen inhibits the 11β-hydroxysteroid dehydrogenase type I isoenzyme activity, reducing the anti-inflammatory effects of cortisol [48], we infer that at low levels of estradiol an intense allergic lung inflammation might be triggered, as observed in OVx-1 protocol. Thus, consistent with the literature, fluctuations of sex hormones during menstrual cycle modifies leukocyte immune function [35], leading to impairment of the inflammatory response during the allergic lung inflammation [15,29]. Our data point out to the relevance of sex hormones status of women (estradiol/progesterone) at the time of sensitization/antigen challenge.

## Conclusions

The clinical implication of this study relies on estradiol and progesterone as modulators of the phenotype of an allergic lung inflammation. Our data contribute to the understanding of mechanisms underlying the deterioration of asthma symptoms in women, clinically observed during and after the fertile phase of the female reproductive cycle.

## Competing interests

The authors declare that they have no competing interests.

## Authors' contributions

APLO peformed the ovariectomy, cell counts, explant cultures, immunostaining and prepared the first draft of the manuscript. JPSP participated in the immunostaining and prepared the manuscript. ADS performed mast cell degranulation assay. ALSF helped carrying out the BAL, blood and bone marrow assays and participated in the preparation of the manuscript. HVD performed cytokines quantification SMO performed mast cell degranulation assay. RMOF corrected the manuscript. BBV co-developed the study idea and corrected the manuscript. WTL developed the study idea, participated in the design of the study and coordinated the experimental work. All authors have read and approved the final manuscript.
